# Digital humanities in the era of digital reproducibility: towards a fairest and post-computational framework

**DOI:** 10.1007/s42803-023-00079-6

**Published:** 2024-01-03

**Authors:** Béatrice Joyeux-Prunel

**Affiliations:** Digital Humanities Chair, University of Geneva, Geneva, Switzerland

**Keywords:** Reproducibility, Digital humanities, Digital art history, Distant reading, FAIR principles, Data

## Abstract

Reproducibility has become a requirement in the hard sciences, and its adoption is gradually extending to the digital humanities. The FAIR criteria and the publication of data papers are both indicative of this trend. However, the question that arises is whether the strict prerequisites of digital reproducibility serve only to exclude digital humanities from broader humanities scholarship. Instead of adopting a binary approach, an alternative method acknowledges the unique features of the objects, inquiries, and techniques of the humanities, including digital humanities, as well as the social and historical contexts in which the concept of reproducibility has developed in the human sciences. In the first part of this paper, I propose to examine the historical and disciplinary context in which the concept of reproducibility has developed within the human sciences, and the disciplinary struggles involved in this process, especially for art history and literature studies. In the second part, I will explore the question of reproducibility through two art history research projects that utilize various computational methods. I argue that issues of corpus, method, and interpretation cannot be separated, rendering a procedural definition of reproducibility impractical. Consequently, I propose the adoption of ‘post-computational reproducibility’, which is based on FAIREST criteria as far as digital corpora are concerned (FAIR + Ethics and Expertise, Source mention + Time-Stamp), but extended to include further sources that confirm computational results with other non-computational methodologies.

## Introduction

During the COVID-19 crisis, there was an increased demand for scientific results to be reproducible and explicable. Some research findings were discredited or subjected to media scrutiny because the research teams were unable to justify their approach. Reproducibility is now considered a fundamental requirement in the so-called hard sciences, and this standard is gradually being adopted by the digital humanities. To validate scientific results, it is expected that the data used should be publicly available, of sufficient and representative size, with transparent metadata, and that the methodology employed should be explicit and reproducible on the data set. The FAIR criteria, which are now required for all research utilizing computational methods, are the cornerstone of the reproducibility principle. Research whose data cannot be found, accessed, interoperable, and reused is deemed non-reproducible. The notion of reproducibility has gained symbolic strength and is increasingly being adopted by research institutions. The advent of data papers in digital humanities is part of the same trend: publication of the code, in addition to making the corpus available, is now required for reproducibility of the method.

This mandate for explicability in digital methodologies within the field of humanities is rather perplexing, particularly when compared to the leniency granted to non-computational research outcomes. The question that arises is whether the digital humanities ought to abide by standards that merely serve to exclude them from the broader purview of humanities scholarship. The crux of the matter lies in a dichotomy: either the stringent prerequisites of digital reproducibility are adhered to scrupulously, leading to an excessively technical discourse that is beyond the comprehension of fellow humanists, or they are not adhered to adequately, leaving the outcomes vulnerable to accusations of being unreliable and, consequently, depriving them of legitimacy in the humanities.

Moving beyond the binary approach, however, there exists an alternative method of considering the matter, which recognizes the distinctive features of the objects, inquiries, and techniques of the humanities, of which digital humanities still form an integral part. The corpora of the humanities are not machine-generated. They are typically diachronic, semantically intricate, qualitative but abundant in quantity, and necessitate an interpretive, even hermeneutic, approach. The issue of reproducibility cannot be examined solely from a procedural perspective. Furthermore, even if the concept of reproducibility were to be precisely defined and explained (…as being reproducible), its implementation has been contingent on particular historical, disciplinary, and societal contexts. Hence, it must also be evaluated from this perspective.

I will therefore first consider the question of reproducibility in the digital humanities from a historical point of view (that of my original discipline), before considering it in the successive stages of the scientific process in the humanities refusing to reduce these stages to mere technical procedures. My argument is two-fold. Firstly, I maintain that within the human sciences, the concept of reproducibility is complex and contextual, and has been wielded against computational approaches mainly when they challenge established disciplinary paradigms. As a result, it is important to recognize the historical and disciplinary context in which this concept has developed. Secondly, I shall delve into the inquiry pertaining to reproducibility within the framework of two research endeavors focusing on the dissemination of artworks and images. These projects employ an array of computational techniques, including quantitative analysis, spatiotemporal visualization, and artificial intelligence. Through their elucidation, I aim to accentuate the methodological complexities inherent in the issue of reproducibility within the domain of human sciences. Indeed, issues of corpus, method, and interpretation cannot be neatly separated, rendering a procedural definition of reproducibility impractical. Instead, I argue that multiple types of reproducibility should be considered, each of which may be more relevant at certain stages of the research process than others. Hence, I put forth the notion of embracing 'post-computational reproducibility' in the realm of humanities, advocating it as a more stringent criterion than the presently endorsed standard. Such an approach holds the potential to mitigate the risk of digital humanities to progressively isolate themselves within the humanities sphere, instead of diffusing their discoveries to a broader and more diverse audience.

## Challenging digital methodologies: the evolution of the reproductivity argument in academic discourse

In the context of contemporary scholarship in the humanities, scholars who utilize computational methodologies frequently encounter inquiries such as "Could you explicate your methodology and sample selection?" As a member of this particular group, which at times feels marginalized within the humanities, I am cognizant that such inquiries often carry an underlying intention to undermine the significance of one's research findings. I posit that the growing inclination within the digital humanities community to emulate computational scientific methods may arise from the distressing experiences endured by some scholars who have faced public rebukes for their adoption of computational approaches. Regrettably, such occurrences appear to be relatively commonplace in certain academic disciplines.

As a consequence, digital humanists have diligently endeavored to establish their credibility and unwavering commitment to the principle of reproducibility. In pursuit of this objective, they have made earnest efforts to disclose their datasets, algorithms, and have even resorted to publishing data papers. Additionally, they have adapted the format of their articles to mirror the structure and appearance of computer science conference publications, meticulously aligning with aspects like length, layout, and Latex formatting. Regrettably, although well-intentioned, these endeavors prove insufficient in justifying their research findings to the individuals who should be the most interested in and benefit from them. This inadequacy stems from the application of visual and linguistic semiotics that remain unintelligible to those in the traditional humanities and the broader public.

As the digital humanities community imposes heightened rigor and technicality in its reporting practices, digital humanities papers tend to become less accessible to colleagues who work with similar corpora but may lack computational expertise. Conversely, digital humanists increasingly neglect to present their findings to specialists in the relevant domain of the corpus, drawn by the allure of establishing themselves within the emerging field of digital humanities, thereby inadvertently isolating themselves from colleagues in their original disciplines. While some celebrate the proliferation of digital humanities conferences, the community should approach this trend with caution. These conferences predominantly function as platforms for digital humanities specialists to engage with one another, fostering exchanges that predominantly revolve around methodological discussions without a profound understanding of the specific corpora being addressed. Digital humanists should also (and instead?) invest time in presenting their findings to colleagues well-versed in the pertinent issues and data, enabling in-depth discussions with individuals knowledgeable about the intricacies of the subject matter.

Remarkably, this transformation is a relatively recent phenomenon. It appears to be primarily evident in two disciplines that have traditionally exhibited a lesser inclination towards computational methodologies: namely, literary studies and art history. In contrast, the fields of history and linguistics seem to be comparatively immune to such approaches.

### History and linguistics: little controversy

Within the field of linguistics, computational approaches have a longstanding history. The inception of computational linguistics dates back to the 1950s, wherein it developed its own unique methodologies, inaugurated its own scholarly journals in the 1960s, and established distinctive modes of inquiry and reporting (Schreibman Siemens Unsworth, 2018). Due to the firm establishment of these methods, there are relatively few debates surrounding them. Additionally, while computational linguistics clearly differentiated itself from classical linguistics, it also provided the latter with valuable corpuses and tools, the practicality of which was swiftly recognised by scholars.^1^ This, in turn, may have deterred them from scrutinising the fundamental methodological underpinnings and the representativeness of the sources from which they derived benefits.

The employment of quantitative methodologies in historical research has an even lengthier tradition, particularly archaeology, a field which necessitates the comparison of disparate sources, often in metric terms, such as number, latitude/longitude, excavation depth, size, and so forth (Grosman, 2016). Students in this area have traditionally received training in quantitative and cartographic techniques. The field of economic and social history, where on the other hand data is abundant, has published quantitative research since the late 1920s, under the auspices of the *École des Annales* (Burke, 1991; Burguière, 2006). The Annales gained acceptance for their findings while maintaining the use of non-quantitativist methods, as exemplified by Fernand Braudel's tripartite approach (Braudel, 1949). Consequently, the utilization of computational methodologies in historical research is generally non-controversial, as long as certain conditions are met. These include making the corpus explicit, ensuring that the analysis does not solely rely on quantitative findings, and critically interpreting the results by corroborating them with other indicators (Zalc & Lemercier, 2008). The latter was the basis for the controversy surrounding certain works in the 1970s, such as Fogel and Engerman's study of slavery in the United States, which were overly reliant on statistical data and lacked critical engagement with the corpus (Fogel and Engerman, 1974; Gutman, 2003). While it is not difficult to publish articles containing quantitative components and graphical or cartographic visualizations in a historical journal, this is not the case in fields such as art history and literature.

### Literary studies and art history v. the calculating mind

In contrast to the fields of history and linguistics indeed, literary studies and art history have been and remain the site of intricate disputes regarding computational methodologies. During these discussions, the issue of reproducibility was not initially a significant point of contention. One notable example is the response to Franco Moretti's application of metric approaches in literary studies (Moretti, 1999 and 2005). It is worth noting that Moretti's methods were not new, nor was his utilization of statistical techniques groundbreaking. Why did his work trigger polemics? Moretti presented his approach as a way of challenging the canon of his discipline, which he did indeed by railing against the inability to move beyond Shakespeare, Corneille, Racine, Hugo, James, Balzac, Dickens, Zola, Joyce or Proust, and to look at something other than the literary piece. This tactic proved effective in provoking outraged reactions from prominent scholars in literature departments at American and European universities during the 1990s and 2000s. They defended style and great literature, quality over quantity, finesse over calculation, diachrony over synchronicity, sophisticated storytelling over simplistic visualization. These initial arguments also effectively countered the symbolic violence of numbers, charts, and maps that Moretti brandished.^2^ However, Moretti was not really questioned about his choice of corpus and methods, and the limitations of his approach went unaddressed.^3^

Certainly, I can utilize my own experience as an illustration within the context of the field of art history. When I presented my doctoral research on the international dissemination of modern and avant-garde art and the careers of artists at art history conferences during the early 2000s, I faced a response that is commonly observed within this discipline, even though my research encompassed not only computational approaches but also other methodologies. A particular set of expressions was recurrently employed, such as "What about the 'work as a work'?" Notably, one of my colleagues made a remark following a colloquium on art history and quantitative methodologies that I had organized in 2008 (Joyeux-Prunel, 2010), stating "on ne met pas la beauté en boîte," which translates to "beauty cannot be boxed." This statement poignantly highlighted the prevailing perception that computational techniques are deemed unsuitable for addressing the interests of a substantial contingent of art historians (and literary specialists), with a specific emphasis on the notion of beauty. The quantitative approach was seen as a misstep, a breach of taste, and a complete misunderstanding of the pivotal concerns within the field of art history.

The computational methodologies posed in fact another significant problem, by challenging certain academic disciplines that regarded themselves as the exclusive authority on the study of creative works. By employing an approach that appeared to be reductive, computational techniques were perceived as a threat. If one were to categorise artists into statistical series, the exceptional cases would disappear, and the brilliance of the artist would be diminished. Tracking the progression of works and highlighting the concurrent evolution of their value was considered problematic, as it risked entangling market, economic, and strategic matters with art. This approach could potentially result in the artist no longer appearing as an independent, autonomous, and isolated figure who is unconcerned with money and success. In short, these new methodologies moved away from the individual work and artist, and instead focused on the collective, which ran counter to the fundamental principles on which these disciplines were founded: the adoration of genius, exceptionalism, and the belief in autonomy from external factors such as economic, political, religious, and social influences.^4^ Of course, it would be incorrect to entirely reject these values. Should we ask a museum to forgo solo exhibitions and admit that the artist they are showcasing is not as great and unique as initially believed? Can we ask a publisher to acknowledge that the author they are publishing imitates the style of a specific writer? The irony, however, is that the reluctance of art history and literary studies to adopt computational methodologies was and continues to be perceived as the final bastion of intellectualism against the influence of market-oriented logic.^5^

Consequently, computational approaches have yet to establish a place in literary studies and art history. It could be contended that established journals in both fields are gradually becoming more receptive to digital methods, which occasionally merit a special issue.^6^ However, these journals seldomly feature an article on digital art history outside of these special issues. In order to publish their unconventional work containing merely a map or a graph, researchers employing computational approaches in art history have been led to establish their own journals.^7^ Similar changes are also occurring in literary studies.^8^ Additionally, in these disciplines, curricula have scarcely been amended to incorporate digital methodologies.^9^ As a consequence, to secure acceptance of a work utilizing computational methodologies, it remains advantageous to publish it without overt indicators of the computational approach. A case in point is my three-volume opus titled "Transnational History of the Avant-Garde," wherein maps and charts were deliberately omitted, despite the foundational reliance on computational methodologies supporting the presented theses (Joyeux-Prunel, 2016, 2017, 2021a, 2021b). Remarkably, the French edition of the inaugural volume witnessed widespread circulation, with 10,000 copies disseminated in 2023. I am inclined to believe that had the book been replete with graphs and predominantly reliant on quantitative methodologies, its reception within the realm of art history would not have been as favorable.

### From opposing 'technopositivist rationality' to attacking methodology: a trial lost in advance for the DH

In 2011, Moretti expressed his lamentation over the absence of a genuine theory that countered Distant Reading. Indeed, the dichotomy between human and machine had become emblematic of the criticisms directed towards digital humanities and digital art history in the late 2010s, and it must be acknowledged that the theoretical potency of this argument is rather feeble.

Of particular significance, however, is the observation that, around 2020, the tide turned: opponents of digital humanities begun to take seriously the question of reproducibility of results, as well as the pertinence of machine usage. In a 2016 article that scrutinized the relationship between literary studies and numerical data, Andrew Piper noted that computational approaches were now routinely expected to outline their methods (Piper, 2016). While Piper concurred that such a demand was appropriate, he was taken aback by the fact that non-computational approaches were not held to the same standard. Since then, a notable shift in attitudes has emerged. Detractors of numerical approaches have begun acquainting themselves with these techniques to more effectively communicate their concerns, thereby fostering a more constructive discourse.

Several instances can illustrate this progression. For instance, art historian Claire Bishop's manifesto titled 'Against the Digital Humanities,' published in 2018, amalgamates both old conventional and new arguments to challenge the constraints of DH (Bishop, 2018). Within Bishop's manifesto, one can observe a dialectical interplay between two distinct arguments. The first argument draws upon the conventional notion that associates computational approaches with neoliberalism, as described in Wendy Brown's book *Undoing the Demos* (Brown, 2017). Following Brown, she alleges that the DH are part of neoliberalism and its alarming technopositivist rationality, a form of reason that is synchronously aligned with the marketization of education (Bishop, 2018, p. 126). Bishop also states that the creation of job positions in DH has been at the cost of the "analog humanities" –as usually, this corporatist viewpoint is not based on specific measures. She then emphasizes the inadequacy of DH approaches to address the interpretive phase of their visualizations, their flawed perception of pure objectivity, their uncritical assumptions about the intrinsic value of statistics, and the tendency to start with the corpus before framing questions.^10^

In addition to the conventional arguments, Bishop introduces new arguments that specifically target the statistical method: 'study that mobilises Big Data needs to reflect critically on *the mechanisms by which this data is gathered*: corporate data mining, state surveillance, and algorithmic governance techniques' (p. 126). While her point is not so much to describe the mechanisms as to reject them for their alleged police taint, the argument does end on the ‘struggle [of these approaches] to explain causality’ (p. 127). Bishop herself utilizes a database for the second part of her article, although she uses it to create ‘hundreds of case studies’ (p. 128).

Undoubtedly, critics of quantitative approaches have undergone a realization that the most persuasive arguments against these methods do not solely emanate from the humanities’ values or political standpoints. Instead, they discern that the crux of the matter resides within the statistical methodology itself. Advocating solely for literary works, esteemed authors, or the pursuit of finesse might inadvertently reinforce the viewpoints of scholars like Moretti. Similarly, dismissing the outcomes of digital humanities research by asserting that no valuable insights are obtained is nor a valid or sophisticated argument.^11^ The proliferation of digital technologies, which are employed by libraries, museums, and archives to enhance accessibility to our cultural heritage, has also necessitated a shift away from the rigid refusal to ‘box’ works of art. Rather than attacking these approaches based on their assumptions and outcomes, it is thus more pertinent to critique them based on the corpus and methods employed. The attack on the corpus, first, happened to be easier. How could Moretti, for example, claim to give access to the 99.99% of the world's literature forgotten by the canon? First, his counts lack certainty and exhaustiveness, rendering them incomplete. Second, these counts focus on bibliometric data that only describe the texts from an exterior point of view. Third, the counts are problematic since they comprise incomplete censuses of different countries that are difficult to compare across nations. As a result, the requirement for exhaustiveness, representativity and explainability, strangely forgotten in most non-computational approaches, suddenly became urgent.

Recently, some scholars have turned to the computational approach to expose the biases, flaws, and limitations of other scholars' work. For example, Katherine Bode argues against Ted Underwood's position on ‘digital evidence’ (Bode, 2020; Underwood, 2019) using statistical theory as her primary argument. Bode challenges Underwood's assertion ‘that statistical analyses can stand on their own,’ and that ‘debates about data "reprentativeness" (…) are resolvable (..) by statistical means.’ She identifies three main problems with Underwood's position:

it misinterprets the scholarly approach to quantitative literary studies; it misconstrues key statistical principles; and its theoretical framework of perspectival modeling neglects critical, political, and ethical issues implicated in using data to understand literature.

Replicability is a critical issue in this debate. Contacting researchers, asking for their corpora, not receiving a reply (especially when the research was done at a time when the FAIR requirement was not widespread), redoing the process and showing that the results would be different -nothing is more effective in denying not only the relevance of a research, but in denying *par ricochet* all relevance to text mining and computational stylistics (Da, 2019). This 'computational case,' initiated by a small group of researchers who have suddenly developed a strong interest in scrutinizing the limitations of their fellow researchers' work, is, in essence, a trial that seems to be predestined for defeat for the accused parties.

### AI, explainability, and the widening gap between digital humanities and humanities

The emergence of deep learning has exacerbated the divide between Digital Humanities and the broader field of humanities, with explicability becoming a touchstone of a deceptive landscape in which DH appears to be largely at a disadvantage.

Returning to the context, during the late 2010s, certain artists and intellectuals have voiced criticism regarding the inherent biases in Artificial Intelligence, particularly within the realm of contemporary art. This critique stems from concerns over the utilization of algorithms trained on flawed and structurally racist datasets for various purposes, including political, law enforcement, military, industrial, and commercial or applications. Trevor Paglen^12^ and Hito Steyerl's work,^13^ along with the *Anatomy of an AI System* by theorist Kate Crawford and artist Vladan Joler (2018),^14^ are well-known examples. In response to this, the art historical community has also spoken out against the black box of 'algorithms', not just in terms of their application to the police or industry on particular datasets, but also with regard to their mere application to art history, -this time, irrespective of the image corpus being used.^15^

In response to the anti-AI political arguments, it seems strategic for those employing machine learning techniques to demonstrate their ability to explain their methodology. DH scholars have increasingly presented more technical approaches, ostensibly seeking to ‘explain’ their methodologies to move away from subjective arguments and establish a foundation based on robust evidence. A growing emphasis on technicality has emerged, often surpassing the expertise of project leaders and compelling them to relinquish their focus on humanities. Within the domain of digital humanities, there exists a demand to mimic computational sciences to an extent that borders on the absurd. If one utilizes AI, scholars may face inquiries about the specific dataset used to train the model (even if they were not the ones who conducted the training). In art history, the question arises very often in conferences, as individuals with even a basic level of digital literacy understand the biases inherent in algorithms that are trained on ImageNET, a contemporary database of photographs tagged with a limited number of content indications of contemporary Western objects and not symbolic objects or artistic forms typically found in historical art. Users are aware of the limitations of these techniques when it comes to classifying reproductions of paintings, sculptures, or prints.

In response, scholars have the option to remind that algorithms are merely tools, and that their outcomes in image classification can be scrutinized, verified or refuted. However, a majority have chosen to align with computer science practices, as if this could provide insights into the effectiveness of the algorithms employed. It becomes crucial for them to demonstrate that algorithms have been utilized in the most efficient and impartial manner possible. Digital art historians might provide classification scores (even though in most cases, their research only retains what has been verified manually, knowing that manual verification is quicker than manual classification). They explain how the algorithm organizes their data, even though they themselves may have only a vague understanding of concepts such as cosine distance, and their primary interest lies in obtaining machine-generated rankings, from which they will select what they deem relevant. A considerable amount of effort can also be devoted to developing or refining specific algorithms, achieved through their training on meticulously curated art historical datasets, distinct from ImageNET or Wikidata. This would ensure accurate image classification based on original mediums, such as distinguishing between drypoint and burin engravings.

Consequently, scholars engaged in projects utilizing machine learning techniques often find themselves investing substantial effort in the presentation of figures, scores, and algorithmic references, which may not directly contribute to their research objectives, can become a distraction, impeding their ability to concentrate on the substantive aspects of their research. In fact, one could argue that this considerable time investment does little to advance their actual research. To be pragmatic, in many classification tasks applied to humanities datasets, an efficient approach involves employing relatively unprocessed algorithms and subsequently manually verifying the classifications. This approach allows for quicker classification while still maintaining a reasonable level of accuracy, as opposed to the laborious process of constructing a highly refined dataset and retraining the model. This pragmatic stance facilitates a more expeditious advancement of research objectives without compromising the overall quality of the outcomes. Moreover, demanding reproducibility for processes involving deep learning algorithms is an exercise in futility, as we are unable to provide explanations for how these algorithms arrive at their outcomes. While we can improve datasets, retrain the machine, and enhance its efficiency scores, the ‘black box’ nature of deep learning remains enigmatic (Offert & Bell, 2021). Even if the corpus is expanded, the biases persist. Therefore, the general outcome is that digital humanities invest significant time in adopting computational rhetoric that fails to convince their critics, while simultaneously preventing them from gaining a foothold in computational sciences, primarily due to a lack of expertise.

### What needs to be reproducible? two cases in art history

As a result, the inclusion of reproducibility requirements in digital humanities research should remain a subject of debate. Adhering strictly to computer science-style reproducibility, which demands complete explanation of the research corpus and computational methodologies, as well as exact replication of results, could result in unwarranted criticism and stifling limitations for computational approaches in the Humanities. Several factors underpin this position, including the recognition that achieving an entirely explicable corpus is a myth, the inherent limitations of reproducibility and perfect explication of methodologies even within strictly computational methods, and the challenge in reducing human sciences' methodologies to mere mechanical procedures. Therefore, the demand for reproducibility, in the computer science sense of the term, is applicable only to specific limited elements of research, linked to a well-defined corpus and limited algorithmic manipulations.

To exemplify this perspective, I will delve into two projects in art history and visual studies that I consider to be representative of digital humanities practices, given their utilization of various digital humanities approaches. In both projects, collective constitution of corpora and source recovery, data restructuring, statistical analyses, and spatiotemporal visualizations are employed. Additionally, on the algorithmic side, the first project employs automatic transcription, while the second project employs computational analysis of images. When analyzing the methodologies of these two projects from the perspective of reproducibility, it is necessary to distinguish two levels: the reproducibility of corpora, and the reproducibility of methods. This analysis reveals that reproducibility can serve to validate a question rather than providing a definitive answer, particularly in the context of larger-scale research endeavors. As a result, I will conclude by proposing a specific approach to reproducibility in digital humanities, and even more broadly in the humanities: a ‘post-computational’ approach that demands the verification of computational results on one corpus through non-computational methods on other corpora, and vice versa.

#### Corpus, 1. data is neither data nor capta; it is a*lea*

Before reproducibility can be achieved, it is essential to establish a corpus. As the same method may not yield identical results when applied to a different corpus, explicability and reproducibility of corpora are crucial touchstones. However, when scrutinizing the question of corpora, one becomes aware that reproducing and explaining them in detail is highly challenging, if not impossible. Hence, let us first examine the corpus of each of the two research projects as illustrative examples.

The first project, Artl@s (https://artlas.huma-num.fr), delves into the globalization of the artistic field and involves the study of the nineteenth and twentieth century global circulation of artworks and artists. To accomplish this, Artl@s provides open access to a comprehensive worldwide database of exhibition catalogues from the nineteenth and twentieth centuries (https://artlas.huma-num.fr/map). Our second example, the Visual Contagions project (https://visualcontagions.unige.ch), extends its investigation to the globalization through images by incorporating a corpus of illustrated periodicals from the years 1890 to 1950. This corpus allows for the study of the international circulation of printed images. In both cases, the research team encountered significant challenges in recovering the necessary digital sources. Apart from the limited accessibility of the original corpora (exhibition catalogues for Artl@s, illustrated journals for Visual Contagions), the teams continually faced input errors, both from the institutions that made the sources available online and during their own encoding processes. Formatting difficulties persisted, despite having clear research questions, such as determining start and end dates or identifying exhibition venues. Lastly, the constitution of the corpora posed serious obstacles and complications when attempting to enrich the recovered data.

For Artl@s, since the early 2010s contributors scattered across the globe have been gathering collections of 19th and 20th-century exhibition catalogues from various regions worldwide.^16^ The collaborative nature of the project means that the augmentation of the corpus depends on the interests of the contributors, and not solely on the concrete history of exhibitions or the presence of a published catalogue for each exhibition. The contributors’ specific research focuses and motivations inevitably result in the corpus being predominantly European, and in some cases, even Parisian. It is challenging to escape the Eurocentrism of art history, even though these catalogues offer a much broader perspective on art history than the traditional museum canon. Additionally, the availability of catalogues is contingent upon preservation and accessibility, which are not always guaranteed outside Western Europe and North America. Once a catalogue is located, the quality of its encoding is reliant on the skills of the contributors. Each transcription is carefully executed within a strict interface, and over the past two years the use of algorithms has aided in the transcription of digitized originals, with regular improvements made to their performance.^17^ Despite this mechanization, which reduces the likelihood of errors, the database may still incorporate erroneous information, such as georeferencing Zurich to Sweden or misidentifying painter Gabriele Münter as male. Even with systematic monitoring, regular proofreading, and support for contributors, it is challenging to obtain a flawless corpus or ensure that no data has been overlooked within the catalogues.

Drawing from my almost 15 years of experience with Artl@s, I recall that in a digital humanities project, sources are not merely acquired or bestowed, nor are they simply ‘taken’ or captured, as is often claimed in reference to Johanna Drucker's striking expression ‘Data is capta’ (Drucker, 2011). This notion of 'captured' is misleading, as it implies a level of precision and responsability in the process that is often absent in the messy reality of building DH corpora. In fact, the process of constructing digital corpora is often characterized by an entropic phenomenon that is in constant flux, making the question of reproducibility seem trivial. In essence, building digital corpora is akin to fishing -one collects what is found, often randomly, and then must make sense of it. Therefore, it can be argued that data is a game of chance. Data is *alea*.

These aleas, encompassing technical, temporal, skill-related, political, and source availability factors, pose numerous challenges. Hence, can one precisely account for a corpus? Beyond the political, geopolitical, economic, legal, and social limitations of data availability, the data is constrained by the structures in which information is formatted; for example, an SQL database imposes more restrictions than an RDF database. Additionally, the process of selecting what to retain or discard, such as introductory texts or illustrations, significantly impacts the quality of content within the corpus. Moreover, the capabilities of individuals involved in encoding the data, along with the accompanying algorithmic frameworks, play influential roles in shaping the corpus. Ultimately, the concept of a corpus is intricate and multifaceted, influenced by a combination of aleas that cannot be fully purified. As a result, it becomes essential to approach corpora from a cross-historical perspective, drawing upon the notion of "Histoire croisée" coined by Werner and Zimmermann (2003), rather than viewing them merely as raw scientific products produced by machines.

#### Corpus, 2. beyond FAIR, we need FAIREST

Instead of attempting to eliminate aleas, the strategy may be to embrace them and incorporate human expertise. This approach becomes particularly apparent in the corpus of the Visual Contagions project. The team, consisting of around ten contributors, has been gathering a substantial collection of digitized illustrated periodicals to investigate the global circulation of images. With a small team, one might expect fewer biases and errors. However, the assembled corpus predominantly focuses on Western Europe and North America and lacks sufficient sources from Northern, Central, and Eastern Europe, as well as Italy, Spain, Portugal, South America, Africa, and Asia. The reasons behind this are manifold. In the Global South, many institutions lack the resources, expertise, or technology for digitizing their cultural heritage. Additionally, the non-standard format of printed materials in some countries complicates the digitization process, necessitating conversion to an interoperable image IIIF format.^18^ Even large institutions, such as the Library of Congress, face difficulties implementing IIIF, leading to restricted access to certain journals. The conversion of digitized materials is time-consuming and requires significant infrastructure and storage space, making it expensive. Moreover, some countries charge for digitizations, even for periods that are in the public domain, further limiting access to visual materials. Another major limitation of the Visual Contagions corpus is image rights, which results in a disproportionate distribution of images chronologically. The corpus predominantly focuses on the period 1890–1950, making it challenging to conduct relevant analyses of the post-1950 period, despite the presence of documents from that era in our sources. In addition to these challenges, there are instances where data that was once available online becomes inaccessible. This can happen when the servers of the institutions hosting the data no longer function or when the data is intentionally removed or altered. Finally, there is a lack of clarity regarding the selection process for illustrated periodicals by the institutions responsible for their online publication, as well as the reliability of the metadata they provide.

Therefore, applying the FAIR principles to a project like Visual Contagions is indeed complex, particularly when the corpus relies on multiple institutions and is not static in its composition. Ensuring that the complete content of the corpus remains F-findable and A-accessible becomes challenging when data availability is not guaranteed or when changes occur over time. Additionally, while making data I-interoperable is important for facilitating R-reuse, it does not automatically guarantee that the corpus will be readily usable by other projects seeking to validate or build upon the analyses conducted. In this context, a FAIREST approach, which goes beyond the FAIR principles, can be advocated. The FAIREST approach entails respecting the FAIR principles as much as possible but also taking into consideration additional factors:

- E for expertise and ethics: Researchers should acknowledge and embrace their expertise in constructing the corpus and maintain ethical considerations in their research.
- S for source-mention: It is essential to clearly document and provide information about the sources used in constructing the corpus.
- T for timestamping: Researchers should acknowledge that the corpus is a snapshot taken at a specific point in time and that it may evolve with new findings or updates.

By adopting the FAIREST approach, researchers acknowledge that constructing a research corpus involves a combination of technical, contextual criteria, and their own expertise and judgment. This approach acknowledges the dynamic nature of research data, embraces transparency in source attribution, and acknowledges the limitations inherent in the corpus construction process. It emphasizes the responsibility of researchers in rendering an account of their corpus and being aware of its context, ultimately promoting a more comprehensive approach to data management and research practices. As for the reproducibility of the corpus, it is now merely a theoretical concept. The paramount concern revolves around its quality, a guarantee that solely human expertise can provide.

### Method, 1. replicating the method?

One might argue that the reproducibility requirement is more applicable to methods than to corpora. The crucial aspect for reproducibility is that researchers can access the corpus to manipulate it and verify the outcomes. Nevertheless, it is uncertain whether the availability of the primary corpus is sufficient for potential censors to replicate our procedures, as in the digital humanities the method modifies the corpus along the way.

Computational approaches in the humanities often focus on sorting and creating sub-corpora indeed. These corpora are the actual corpora studied, through procedures that require numerous micro-decisions that are challenging to trace. For example, Visual Contagions' primary objective is to identify images that have been reproduced several times or imitated from a global corpus. Before that stage, we require algorithms to isolate and segment the periodical illustrations in the primary corpus, which are then grouped by visual similarity. The segmentation algorithm restores the coordinates of each illustration in the pixel matrix of a periodical's page, allowing us to reduce each illustration to a vector that is projected into a specific geometric space using Principal Components Regression. This enables us to recover batches of images based on visual similarity. Each image inherits the date, title, place, and type of publication from its medium. It is therefore possible to track batches of similar images in space and time, based on the clusters recovered by comparing illustrations. So far, the process seems easy to reproduce. However, using the same algorithms would not be enough to reproduce the approach: they need to be parameterised, and these parameterisations have been often intuitive.

Why did we choose this algorithm over another? Only because the results it provided were more in line with our expectations -grouping images by similarity, without preventing the recovery of exact duplicates. First, we built the vectors summarising each image (features) using a widespread network, ResNet18, itself trained for classification on ImageNet, with 11 million parameters, and without reprocessing the dimensions of the vectors. The results were disappointing: when searching for known and widespread images in the corpus, the network only returned a small number of duplicates. From trial and error, we settled on the ViT network and the DINO training method: the results were more suitable, and the computation time was reasonable (Champenois & Joyeux-Prunel, 2023).Thus, from a rational point of view, one could say that we adapted the method to the results we wanted; a very questionable approach.

This attitude can be justified by pointing out that the ambition was not to make an analysis, but to better target our corpus. It is here, in any case, that the method modifies the corpus. Depending on one's point of view, one may consider that it biases it further, or on the contrary, that without it we would have a much less interesting and representative corpus. From the pessimistic point of view, the procedure misses certain images -on the one hand, the segmentation is done with an algorithm whose recall is only 92/93%; which means, since we have recovered nearly 12 million images for the moment, that we are missing at least 840,000 images; on the other hand, some images that are similar to others are missed because, during the principal component analysis that allows the vectors summarising the images to be grouped together, some vectors are too close to several clusters at the same time and end up in an insignificant large cluster. We rerun the algorithms on this snowball, and recover new duplicates, but still lose images. From the optimistic point of view, many images are found. However, this means only the start of real research. The machine tends to group similar images together, but the significance of their similarity can vary depending on the context, making it necessary to inject meaning into the process in order to avoid nonsensical results. For instance, if we were to rely solely on the machine, the barcodes of scanned images would be included in our statistics and analyses, forming impressive international clusters that circulated globally during the entire period. Therefore, we need to take a different approach, where the machine merely suggests, and human experts validate the formation of new corpora. The machine proposes, we dispose. Only after human verification of valid groupings can any data be considered useful for the study. In this way, the responsibility of researchers in the validation and rejection of machine-generated results is essential for the project's success. As for the reproducibility of this initial method, an endeavor to engage with it might prove to be a daunting task.

### Method, 2. The code is not the method

Let us then acknowledge the non-reproducibility of the initial corpus in projects such as Visual Contagions, and the inherent challenge in providing a comprehensive account of the final research corpus formation process. Let us also embrace the notion that certain groups of images, curated based on machine-generated similarities and deemed relevant by expert historians, can be selected to investigate the global circulation of images. Providing accessibility to the curated corpus for methodological verification becomes crucial during the stage of statistical analysis. However, the question arises whether reproducibility of results alone is sufficient to ensure the overall quality of the research.

Let us stay with the example of Visual Contagions. After verifying thousands of groups of similar images manually, we aim to examine the geography of these image circulations and their distribution patterns. Specifically, we are interested in identifying the most influential journals involved in visual circulation, both as importers and exporters. To automatically obtain an overview of these circulations, we encode the images (firstly machine-grouped, then selected by us) into chains of circulation, merge them, and analyse them statistically. This is achieved by organising the objects of each chain in RDF format, which is highly flexible and allows for the addition of new images to related chains. RDF also enables us to define rules for tracking chains of traffic based on relationships and order, such as the chronological ranking of similar images. With this structure in place, we can use algorithms to extract the periodicals that are most frequently present at the start of a chain from all the chains. This process is carried out in a Python notebook, which allows us to evaluate metrics to determine which journals, for example, have initiated more circulation chains than others, or which images have crossed more countries than others.

The approach used here is reproducible, which is both good and necessary. However, I do not believe that the argument in favour of reproducibility lies in proving and guaranteeing the relevance of the results. The code is not the method. Instead, the strongest argument in support of reproducibility is that it allows for iterative improvement of the analysis process.

### For a post-computational reproducibility

If the aim is to ensure the relevance of a result, an approach must be justified by a different type of reproducibility that I refer to as post-computational reproducibility, particularly in Digital Humanities, as opposed to a Cultural Analytics approach, for instance. Indeed, the research process does not end with the numerical analysis of results. Instead, it marks the beginning of an inquiry that generates questions that can be developed into hypotheses. These questions and hypotheses must be corroborated with other sources, analyzed through different methods, and examined on different scales.

For instance, the Visual Contagions project's computational results reveal that artistic images circulated globally more frequently than other images, while images from daily press were more domestically and nationally circulated.^19^ This outcome solely relies on the specific corpus utilized and may vary when the corpus changes. Even though the corpus is the largest, most global, and covers the longest period ever analyzed, the outcome’s validity can only be confirmed when corroborated with other perspectives and additional, different sources such as period testimonies, cartoons, archives, etc.

Similarly, it is possible to conclude from our computational studies that the avant-garde magazines from the 1920s circulated international art images more widely than others, and that Parisian magazines were not as central as they are commonly believed to be in the global history of the avant-garde. The corpus of the study is broad, if not exhaustive, extending beyond what one researcher can study alone. However, the credibility of the thesis can only be substantiated if it is authenticated by other sources, such as the opinions of observers from the period, artists' career trajectories, and artwork examination.^20^ Therefore, the epistemological status of a computational approach result can only be a question or a supplementary argument in a broader inquiry.

If the outcomes of a computational investigation are not corroborated on other levels and with diverse sources, it may be necessary to modify the corpus or the methodology, despite being reproducible. This is precisely the challenge with Cultural Analytics, whose primary utility is to open new inquieries, not to provide conclusive answers, even though their results are frequently taken at face value –maybe because no further analysis is tempted beyond statistics (Manovich, 2020). Can we assert that we are studying global historical developments by relying on Google N-Grams? Or that we can explain the evolution of cinema in the twentieth century with a vast collection of films? Or that ‘notable’ artists were born first in Europe, then in America, by relying on the entire corpus of Wikidata and thus verifying the conventional thesis of *translatio imperii* from Florence and Rome to Paris and New York?^21^ Although the method is transparent, the analysis process is faultless, and the corpus is unblemished, what can the approach assert? Essentially, nothing concrete, except that ‘such and such’ is occurring on this limited and prejudiced corpus. It is evident that most of the time, the ‘such-and-such’ is happening since the corpus is structured and filled in a particular way. For instance, Wikidata is not a source of the past; it is a source of how we discuss and conceive the past today. Therefore, it is unsurprising that Wikidata reflects the idea of a *translatio imperii*. If we study the same question on other levels, on specific corpora taken from the past, such as exhibition catalogues, or examine artists who did not create work in the centres in question, particularly if we consider domination not solely as a history of quantity but as a history of perception, we will not arrive at the same conclusions (Joyeux-Prunel, 2015 and 2021a).

The ability to replicate a computational approach is undoubtedly valuable, yet it alone is inadequate to support excellence of research. Rather, the challenge lies in producing more representative outcomes that can be validated using other data sets and on a larger scale. In the realm of digital humanities, it is imperative that reproducibility is not limited to the computational results produced via a single methodology and a single data set but is extended to include both computational and non-computational methods across various data sets.

The post-computational approach goes beyond distant and computational methods, as well as all scholars should go beyond limited subjectivity confined to supposedly selected case studies. Instead, it acknowledges the critical role of human interpretation in comprehending research results, while recognizing that this understanding requires substantial elements—their solidity determined by experts (where a graph may be less robust than an analysis grounded in period documents). By diversifying and expanding the scales of analysis, researchers can strengthen the validity of hypotheses originating from one level by corroborating them with findings from different levels of inquiry. For instance, global quantitative approaches' hypotheses should be complemented by diverse sources and analyzed using varied methodologies across multiple scales. Reciprocally, enriching case studies through broader contextualization bolsters their resilience and uncovers valuable insights. Embracing an insatiable thirst for knowledge, researchers should persistently seek fresh perspectives, remain unsatisfied with isolated findings, and foster collaborative endeavors to ensure a both holistic and more precise grasp of intricate phenomena. The idea of a “Post-Computational Reproducibility" also encourages researchers to explore how various scales of analysis interact with each other to strengthen the reliability of research outcomes. By illustrating the iterative nature of research, where findings are continually challenged, refined, and expanded upon, researchers can emphasize the importance of collaborative efforts and multidisciplinary approaches.

Post-computational reproducibility involves integrating diverse sources of evidence that corroborate a single hypothesis, which, in turn, enhances the credibility of research. The aim is to persuade not just computer scientists, but also those who are interested in our research subjects. The ultimate prize is that the outcomes of our work endure, and that the hypotheses generated via the computational approach are further tested and deemed convincing by the standards of non-statistical approaches. Otherwise, our efforts risk being relegated to oblivion.

## Conclusion. the DH and their aura in the age of digital reproducibility

In conclusion, it is evident that in the field of digital humanities, a hasty and computerized approach to the issue of reproducibility can pose a significant threat to research projects. Those who are skeptical of computational approaches have used the argument of non-reproducibility to discredit them. However, reproducibility requirements in the digital humanities are only suitable for specific algorithmic stages and will never apply to the corpora. This is because digital humanities data is not pre-determined or captured but instead is initially random. Neither *data* nor *capta*, DH data are rather *alea*.

However, this fragility is not a problem if the corpus is associated with expertise, linked to sources and research, and time-stamped, especially if the corpora evolve with the research. Instead of the FAIR principles (findable, accessible, interoperable, and reusable), the digital humanities thus require a FAIREST approach. This means ensuring ethical corpora that acknowledge expert contributors, sourced to allow a return to the source, and time-stamped if the corpora evolve with research. The reality is that as researchers we should not be satisfied with results that are solely derived from computational methods, regardless of the solidity of the computer procedure used. If the digital humanities hope to establish the legitimacy of their findings, they need to move beyond a reproducibility that merely mimics that of computer science, otherwise they would isolate themselves from other human sciences, in particular for literary studies and art history. To resist this tendency, digital humanities researchers must demonstrate the relevance of their computational findings on a broader scale, with other corpora, and using non-computational methods. Post-computational reproducibility is socially beneficial for the discipline since it encourages researchers to engage in scientific responsibility, prevent them from becoming reliant on machines, and enable them to retain their expertise. Failure to do so could lead to a loss of interest from other human sciences, causing digital reproducibility in digital humanities research to lose its aura, similar to Walter Benjamin's analysis of art in the age of mechanical reproducibility almost a century ago (Benjamin, 2015).

## Footnotes

1 For example, for the French language: Frantext, created as an ‘electronic database’ in the early 1960s, published online in 1998 (See Martin, 1991). For English: the British National Corpus: https://www.english-corpora.org/bnc/ (“created by Oxford University press in the 1890s-early 1990s” – homepage consulted 17 March 2023) or the American National Corpus (ANC): http://www.anc.org, “produced from 1990 onward” (homepage consulted 17 March 2023).

2 On the “symbolic violence” see Bourdieu, 1970.

3 For example Goodwin & Holbo, 2011.

4 For example by Krauss & Bois, 1990.

5 In the introduction to Joyeux-Prunel, 2016 (English translation in progress), I expound upon this particular ideology. See also, for literature, Bloom, 1997.

6 Some examples in the field of art history: *Art Bulletin* addresses the issue as early as 1997 (Cohen, K., et al., 1997); but does not appear to have devoted a special issue to the subject. The *Zeitschrift für Kunstgeschichte* published half an issue in 2015 (79. Bd., H. 2, 2016). The journal *Histoire de l'art* published a special issue in 2021 (87. Bd., H. 2*,* 2021/1 (contents: http://blog.apahau.org/parution-histoire-de-lart-87-humanites-numeriques/). On the other hand, there is no special issue devoted to digital approaches in *Art History* (https://arthistoryjournal.org.uk/issues/).

7 *Artl@s Bulletin* was created in 2012: https://docs.lib.purdue.edu/artlas/. *DAHJ—The Platform For Digital ART History was* created in 2015: https://dahj.org/.

8 *Digital Literary Studies* was established in 2016: https://journals.psu.edu/dls. The *Journal of Computational Literary Studies* (JCLS) released its first issue in 2022 (https://jcls.io/).

9 For the field of art history, in a debate published in 2015 Johanna Drucker pleaded for what still seems to be a dream in the USA. Johanna Drucker**, **Anne Helmreich**,**Matthew Lincoln et Francesca Rose**,** ‘*Digital art history*: The American Scene’, *Perspective*, 2 | 2015, DOI: https://doi.org/10.4000/perspective.6021.

10 ‘Moretti-like Lev Manovich-proceeds with the data set in advance of a research question’; Bishop, 2018, pp. 126–127.

11 Well seen for example by Bishop, 2018, pp. 126–127.

12 Cf. the exhibition *Trevor Paglen: Opposing Geometries*; September 4, 2020-March 14, 2021, Carnegie Museum of Art**.**

13 A retrospective point of view in: "Hito Steyerl on Why NFTs and A.I. Image Generators Are Really Just 'Onboarding Tools' for Tech Conglomerates. "This is the Future" has just opened at the Portland Art Museum. Interview with Kate Brown, March 10, 2023. https://news.artnet.com/art-world/these-renderings-do-not-relate-to-reality-hito-steyerl-on-the-ideologies-embedded-in-a-i-image-generators-2264692. Accessed 16 March 2023.

14 MoMA ("Gift of the Designers", 2019). https://www.moma.org/collection/works/401279?artist_id=131890&page=1&sov_referrer=artist.

15 I refer in particular to the theses presented by Amanda Wasielewski, forthcoming in *Computational Formalism: Art History and Machine Learning* (forthcoming May 2023, MIT Press).

16 On the data encoding process see Joyeux-Prunel, 2018.

17 We use the infrastructure FoNDUE (see https://www.unige.ch/lettres/humanites-numeriques/recherche/projets-de-la-chaire/fondue).

18 See https://iiif.io/.

19 These results are presented on the musée du Jeu de Paume’s Space for Digital Creation: https://jeudepaume.org/evenement/contagions-visuelles/, and https://jdp.visualcontagions.net/EN_HOMEPAGE.html.

20 Forthcoming: Béatrice Joyeux-Prunel and Nicola Carboni, ‘Plotting the Geopolitics of 20th-c. Modern and Avant-Garde Illustrated Periodicals. Distant and Close Viewing and the Issue of Centres and Peripheries,’ *Journal of European Periodicals Studies.*

21 This is one of the main issues in Schich, 2016, itself a *résumé* of Schich et al., 2014.

## Declarations

### Data availability

Visual Contagions: https://visualcontagions.unige.ch/explore

Artlas: https://artlas.huma-num.fr/map
